# Arctic Greening Drives Changes in the Diet and Gut Microbiome of a Large Herbivore With Consequences for Body Mass

**DOI:** 10.1002/ece3.71731

**Published:** 2025-07-22

**Authors:** Stefaniya Kamenova, Steve D. Albon, Leif Egil Loe, R. Justin Irvine, Rolf Langvatn, Galina Gusarova, Eric Jacques de Muinck, Pål Trosvik

**Affiliations:** ^1^ Department of Biosciences, Centre for Ecological and Evolutionary Synthesis University of Oslo Oslo Norway; ^2^ Departments of Ecology and Natural Resource Management Norwegian University of Life Sciences Ås Norway; ^3^ National Museum of Natural History Bulgarian Academy of Sciences Sofia Bulgaria; ^4^ The James Hutton Institute Aberdeen UK; ^5^ Frankfurt Zoological Society Markus Borner Center Arusha Tanzania; ^6^ Independent Researcher Trondheim Norway; ^7^ The Arctic University Museum of Norway UiT—Arctic University of Norway Tromsø Norway; ^8^ Department of Botany St Petersburg State University St Petersburg Russia; ^9^ Department of Pharmacy University of Oslo Oslo Norway

**Keywords:** 16S amplicon sequencing, climate change, DNA metabarcoding diet analysis, herbivory, *Rangifer*, rumen microbiome, tundra

## Abstract

Rapid climate warming is ‘greening’ the tundra, reflecting a higher plant biomass, particularly of deciduous shrubs and grasses. However, the consequences of these changes for herbivore nutrition are unclear. Although, the gut microbiome mediates nutrient metabolism, and hence herbivores' capacity to adapt to dietary change, few studies have investigated the effect of annual changes in the diet‐gut microbiome nexus on fitness‐related traits. In a model system, the Svalbard reindeer, a species experiencing the greatest rate of climate warming on Earth, we investigate how changes in diet and the gut microbiome impact reindeer body mass. Using high‐resolution DNA metabarcoding, we assessed diet and gut microbiome bacterial composition in rumen samples from animals culled in October from 1998 to 2004 in four different valleys. Overall diet diversity and grass relative reads abundance (RRA) were significantly higher following warmer summers, while the RRA of the dwarf shrub *Salix polaris* increased with the maximum normalised difference vegetation index (NDVI), our measure of annual biomass. Autumn body mass, a strong proxy of fitness in Svalbard reindeer, was significantly, positively correlated with *Salix* RRA, most pronounced in females that had reproduced, and by that depleted body reserves. Multivariate analyses revealed a highly significant relationship between diet and microbiome composition at the individual level. This included a significant positive correlation between *Salix* and bacterial diversity. However, a structural equation model revealed that the direct path effect of *Salix* on reindeer body mass was stronger than the indirect path effect, mediated through the gut microbiome. Our results suggest that climate‐driven Arctic greening may have implications for herbivore body mass, operating through a change in diet composition. These findings reveal some of the mechanistic underpinnings of Arctic warming on herbivore populations and highlight the importance of the diet−gut microbiome nexus in facilitating species resilience to rapid climate change.

## Graphical abstract

Using high‐resolution DNA metabarcoding to quantify both diet and gut microbiome composition, we test the hypothesis that climate‐driven Arctic greening positively influences reindeer body mass through a gradual improvement in diet, accompanied by corresponding shifts in the gut microbiome. Our results show that the proportion of polar willow (*Salix polaris*)—the dominant dwarf shrub species in Svalbard—increases with the Normalised Difference Vegetation Index (NDVI) and has a significant positive effect on autumn body mass of lactating female reindeer.

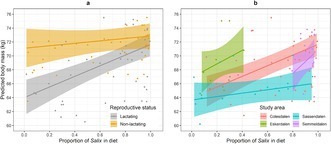

## 1 Introduction

The Arctic tundra is warming fast, with temperatures rising almost four times more rapidly than the global average (Rantanen et al. 2022). Such a dramatic change is already disrupting the ways species interact with each other, with consequences for tundra ecosystem functioning (Post et al. 2009). The resulting warmer and longer plant growing seasons across the circumpolar Arctic have led to widespread increases in plant productivity (Sturm et al. 2001; Myers‐Smith et al. 2011; Forbes et al. 2017; Berner et al. 2020). Many of the plant species benefiting from the warming climate, including graminoids (grasses, sedges) and deciduous shrubs (e.g., birch and *Salix* dwarf shrubs), are the primary forage grazed by keystone large herbivores, such as caribou/reindeer and muskox (Bråthen and Oksanen 2001; Bjørkvoll et al. 2009; Larter and Nagy 2004; Thing et al. 1987). While the quantity of forage plants is generally increasing, short‐term warming experiments suggest plant quality (digestibility and nutrient content) could decline (Zamin et al. 2017), though there may be some acclimation (Leffler et al. 2022).

To date, evidence of the impact of Arctic greening on herbivores is scarce and suggests both positive and negative effects. For example, many circumpolar *Rangifer* populations have undergone marked declines in population size (Vors and Boyce 2009; Fauchald et al. 2017), mainly due to the combined effect of anthropogenic pressure and climate‐driven changes in the sea ice cover, leading to decreased pasture quality and an increase in the frequency and severity of extreme weather events, such as rain‐on‐snow (Fauchald et al. 2017; Forbes et al. 2016). However, on a more local scale, earlier vegetation green‐up and higher plant productivity can increase calf autumn body mass and female reproductive success (Tveraa et al. 2013). Similarly, higher plant productivity in warm summers (Van der Wal and Stien 2014) increases autumn body mass, leading to higher ovulation rates in Svalbard reindeer (Albon et al. 2017). Interestingly, while muskox population growth in West Greenland also benefited from earlier green‐up outweighing the impacts of winter conditions, this was not the case in a sympatric caribou population where numbers declined because spring green‐up seemed to have little impact on calf production compared to winter temperature (Eikelenboom et al. 2021). Thus, the effects of Arctic greening on large mammalian herbivores might be both species‐ and context‐dependent, raising the question about the specific niche requirements and mechanisms underpinning the direction of impacts.

Since ruminant herbivores are particularly dependent on their foregut microbiomes for harvesting energy from the plants they ingest (Leser and Mølbak 2009; Shabat et al. 2016), it would seem prudent to also study the response of the gut microbiome to climate‐induced changes in diet. Typically, the gut microbiome is a highly plastic, rapidly evolving system (Candela et al. 2012). Thus, for ruminant species with long generation times (i.e., little capacity for evolutionary adaptation) gut microbiome plasticity could be a key mechanism enabling rapid ecological adaptation to change in the availability of forage plants (cf Alberdi et al. 2016; Clayton et al. 2016). Nevertheless, only now are we starting to comprehend the links and the feedback between foraging, the gut microbiome, and fitness‐related traits in wildlife (Worsley et al. 2021, 2024; Risely et al. 2023), and the empirical validations remain limited. For example, recent findings from the giant panda show that changes in diet composition and quality could elicit an adaptive response of the gut microbiome, with a direct positive effect on the maintenance of body mass and condition (Huang et al. 2022), while Stothart et al. (2024) found that differences in gut microbiome composition were associated with the probability of survival in Sable Island feral horses. However, so far, long‐term studies on the diet‐gut microbiome nexus have been restricted to humans, mainly showing a positive relationship between diet quality and gut microbiome diversity and functioning (Ma et al. 2022; Yu et al. 2021; Petrone et al. 2023). On the other hand, some studies of the human gut microbiome have shown marked resilience to changes in diet (Fragiadakis et al. 2020), with some bacterial strains persisting for decades (Faith et al. 2013). With the occurrence of such antagonistic processes and the limited set of studies to date, identifying the mechanisms driving the impact of Arctic greening on large herbivores remains a challenge.

Here, in a study of Svalbard reindeer, we address the question of how climate‐driven changes in plant productivity (Van der Wal and Stien 2014) influence the autumn diet and rumen microbiome, and the impact on individual reindeer body mass, underpinning its sustained population growth (Le Moullec et al. 2019). For this, we use 7 years of female reindeer rumen samples and body mass data, collected in late October, when body condition peaks. Using high‐resolution DNA metabarcoding to assess both diet and rumen bacterial composition, we document annual variation in the diet and gut microbiome. Given the increasing plant productivity as summers warmed on Svalbard (Van der Wal and Stien 2014), we test the hypothesis that the estimated relative reads abundance (RRA) of preferred forage plants (graminoids and dwarf shrubs) in the autumn diet increases when there is more forage following warmer summers. Also, we predict a significant positive correlation between these preferred forage plants and autumn body mass, a key fitness‐related trait driving ovulation rates in Svalbard reindeer (Albon et al. 2017), and successful calving the following summer in caribou (Cameron et al. 1993; Gerhart et al. 1997). Finally, given the documented importance of the gut microbiome for both nutrient acquisition and host fitness (Suzuki 2017; Gould et al. 2018), we expect a significant correlation between diet and gut microbiome composition, as well as an independent positive gut microbiome‐driven effect on reindeer body mass.

## 2 Material and Methods

### 2.1 Study Area

Svalbard reindeer were sampled between 1998 and 2004 from two main areas in Svalbard—the Colesdalen and Semmeldalen valleys in Nordenskiöld land (77°50′–78°20′ N, 15°00′–17°30′ E), and the Sassendalen and Eskerdalen valleys in Sassen‐Bünsow Land National Park (78°23′ N, 17°15′ E; Figure S1). The generally wide, U‐shaped valleys are mostly vegetated up to about 250 m altitude, although above‐ground live vascular plant biomass averages only c. 35 g m^−2^ (annual range 23–46 g m^−2^, Van der Wal and Stien 2014). In the drier *Luzula* heath and ridge habitats (c. 40% of the area) dwarf shrub species, particularly *Salix polaris* , account for 61% and 78%, respectively, of the above‐ground biomass in early August. On the less well‐drained shallow slopes and marsh areas, grasses, sedges, and rushes are dominant (Van der Wal and Stien 2014). The percentage of the different habitat types in each valley is presented in Table S1. The period 1998–2004 was an early phase of warming on Svalbard with higher plant productivity (Van der Wal and Stien 2014) and a commensurate increase in autumn body mass of reindeer (Albon et al. 2017). During the study, the estimated reindeer population size in the Colesdalen/Semmeldalen (see Lee et al. 2015) varied between 948 (95% CI: 898–1006) and 1339 (95% CI: 1251–1440) individuals (estimates include lower Reindalen, Albon et al. 2017), while the Sassendalen/Eskerdalen population counts increased from ~750 to ~1250 (Hansen et al. 2019). Populations from the Colesdalen/Semmeldalen and the Sassendalen/Eskerdalen areas are effectively isolated from each other, with little interchange revealed by genetic analysis (Côté et al. 2002).

### 2.2 Sample Collection

Female reindeer were culled in October (between 19 and 27) and body mass recorded before evisceration as part of an earlier study of the impact of gastrointestinal parasites on body mass and fertility (Albon et al. 2002; Stien et al. 2002). At this time of the year, shortly after the rut, female *Rangifer* are heaviest and fattest (Thompson and Barboza 2017). Between 300 and 500 mL of rumen content was collected from multiple locations from female reindeer 2 years and older and stored at −20°C. Individuals were aged by counting rings in the cementum of the first incisor (Reimers and Nordby 1968). A female was defined as lactating if *corpus rubrum* was present in the ovaries (Langvatn et al. 1994) or if dissection of the internal tissue of the mammary gland revealed milk (lactating, *N* = 49, or not lactating, *N* = 48). All samples were collected with the permission of the Governor of Svalbard (Sysselmannen) and met ethical requirements of the Research Council of Norway.

### 2.3 Environmental Variables Measurements

Daily meteorological measurements were recorded at Svalbard Airport, Longyearbyen (Station SN99840, Norwegian Climate Services: https://seklima.met.no/observations) c. 40 km from the most distant valleys. Mean July temperature for the 7 years of this study was 6.93°C ± 0.8SD and showed no temporal trend (*r* = 0.052, *p* > 0.9).

As an indicator of greening, we used the annual maximum remote‐sensed normalised difference vegetation index (NDVI), widely accepted as a correlate of plant productivity (Pettorelli et al. 2011), in particular sensitive to the expansion of shrub species (Jespersen et al. 2023). Maximum NDVI was calculated from 3rd generation Normalised Difference Vegetation Indices (NDVI‐3G+) available from the Global Inventory Modeling and Mapping Studies (GIMMS) dataset of Advanced Very High‐Resolution Radiometer (AVHRR) images at the National Centre for Climate Research, Boulder, Colorado, USA. NDVI values range from +1.0 to −1.0. Areas of snow, rock, or sand show very low NDVI values (0.1 or less). Sparse vegetation such as shrub tundra results in moderate NDVI values (0.2–0.5). In Svalbard, NDVI values peak in late July/early August, coinciding with peak biomass (Karlsen et al. 2018, 2020). The maximum NDVI was estimated from six 15‐day composites from late July to early September each year using pixels of 0.0833° spatial resolution within the Colesdalen/Semmeldalen part of our study area. The mean of the annual max NDVI was 0.226 ± 0.044SD and increased significantly over the 7 years (NDVI, *r* = 0.776, *p* = 0.04), variation which was highly correlated (*r* = 0.993, *N* = 7, *p* < 0.001) with values for a larger area around Isfjorden (Vickers et al. 2016).

### 2.4 DNA Metabarcoding Diet Analysis

Approximately 15 g of wet rumen material was homogenised in liquid nitrogen. DNA was extracted from 100 mg of the resulting ground rumen powder using the NucleoSpin Plant II (Macherey‐Nagel, Germany) and following the manufacturer's instructions. Blank extractions (ultra‐pure Milli‐Q water instead of DNA) were included for every 24 samples. We targeted the plant part of the reindeer diet by using the *Sper01* primers amplifying the chloroplast trn*L* P6 loop in seed plants (Spermatophyta) (Taberlet et al. 2007). This is a commonly used DNA metabarcoding plant marker for which an extensive DNA reference database for the Boreal and Arctic regions is available (ArctBorBryo, Voldstad et al. 2020). PCR amplifications were carried out in a final volume of 15 μL, using the AmpliTaq Gold 360 PCR Master Mix (Thermo Fisher Scientific, USA), 2 μL of DNA extract as template, 0.4 μL of BSA, and 0.5 μM of each primer. The PCR mixture was denatured at 95°C for 10 min, followed by 35 cycles of 30 s at 95°C and 30 s at 52°C, and an elongation step for 7 min at 72°C. An 8–9 nucleotide sequence tag was added on the 5′ end of each forward and reverse primer, resulting in a unique tag combination for each PCR product in order to allow the assignment of sequence reads for the relevant sample. Each PCR reaction was carried out in triplicate and PCR negative controls (ultra‐pure Milli‐Q water instead of DNA) were systematically included to monitor for contamination. We also included one positive control for each 95 PCR replicates. Positive controls consisted of an artificially assembled mock community containing a mixture of varying proportions of six unique synthetic DNA stretches with varying GC content, homopolymers, and sequence lengths (https://github.com/pheintzman/metabarcoding, Table S2). A subset of PCR products was selected for the visual inspection of the amplified DNA using 1.5% gel electrophoresis. PCR products were pooled and purified using the QIAquick PCR Purification Kit (Qiagen, Germany). DNA concentrations from purified amplicon pools were then quantified using a Qubit 2.0 fluorometer and the dsDNA HS Assay kit (Invitrogen, Life Technologies, USA), and pooled again prior to library preparation and sequencing. Libraries were prepared using the KAPA HyperPlus kit (Kapa Biosystems, USA), and sequenced on a HiSeq 4000 machine (Illumina, USA) following the manufacturer's instructions at the Norwegian Sequencing Centre (https://www.sequencing.uio.no). A total of 150 nucleotides were sequenced on each extremity of the DNA fragments.

### 2.5 Bioinformatic Analyses

Sequences were analysed using the OBITools pipeline (Boyer et al. 2016). The direct and reverse reads were aligned and merged using the *illuminapairedend* command by considering the quality of the sequence data during the alignment and the consensus computation. Only alignments with scores > 50 were kept for further analyses. Primers and tags were identified using the *ngsfilter* command. Only sequences with a perfect match on tags and a maximum of two errors on primers were retained for further analyses. Primers and tags were cut off at this step. Strictly identical sequences were clustered together using the *obiuniq* command, while keeping the information about their distribution among samples. All sequences shorter than 10 bp and/or occurring at ≤ 10 reads were excluded. Sequences corresponding to PCR and/or sequencing errors were labelled to be removed using *obiclean*. Taxonomic assignments were carried out using the *ecotag* command and the ArctBorBryo plant reference database. A unique taxon was assigned to each sequence with taxa corresponding to the last common ancestor node in the National Center for Biotechnology Information (NCBI) taxonomic tree. If several matches between the query sequence and the reference database were possible, the sequence was assigned to the taxon corresponding to the last common ancestor node of all the taxa in the NCBI taxonomic tree that best matched against the query sequence. Datasets were imported in R (version 4.4.1) for further curation. Molecular operational taxonomic units (MOTUs) labelled as PCR errors were filtered out. PCR replicate outliers (likely corresponding to non‐functional PCR reactions) were also discarded. For this, we calculated the Euclidean distances of PCR replicates with their average (hereafter *dw*) and compared it against the distribution of pairwise dissimilarities between all average samples (hereafter *db*). Based on the expectation that PCR replicates from the same sample should be more similar than any two average samples (*dw*<*db*), we discarded PCR replicates lying outside the dissimilarity threshold defined as the intersection of *dw* and *db* distributions. This process was repeated iteratively until no more PCR replicates were removed from the dataset. If only a single PCR replicate per sample was left at the end, the sample was removed from the dataset. At the end of this procedure and in order to give equal weight to each replicate, remaining PCR replicates were averaged for each sample. Sequence reads abundance was normalised by dividing the number of reads for each MOTU by the total number of reads for each sample. MOTUs with relative abundance < 1% in at least one sample as well as MOTUs with a best‐identity match below 94% were discarded. Finally, PCR amplification success, tag jumps and potential cross‐contaminations among samples were assessed by inspecting the detection levels and sequence reads abundance patterns of the synthetic standard sequences used as PCR positive controls. MOTUs were manually checked against the Svalbard flora checklist (http://svalbardflora.no) and taxonomic identifications refined whenever possible. MOTUs were grouped into 14 family groups across which we calculated diet diversity (see Section 2.7 below). In addition, we aggregated these family groups into eight functional categories based on Bråthen et al. (2007). Since the proportion of sequence reads assigned to a family group or a functional category is not the same as quantifying intake (Deagle et al. 2019), we compared RRA assigned with DNA metabarcoding with the proportions estimated from published micro‐histological analysis of plant fragments for 64 of the 97 rumens analysed here (see Bjørkvoll et al. 2009). The significant correlations we show in the Supporting Information (Figure S2) demonstrate that it is possible to describe individual variation in components of the diet.

### 2.6 Microbiome 16S Amplicon Sequencing

Library preparation for DNA sequencing was carried out as previously described (de Muinck et al. 2017), targeting the V4 region of the 16S rRNA gene with the 515f‐805r primer pair. 2 × 300 bp paired‐end sequencing was performed using the MiSeq platform at the Norwegian Sequencing Centre, Oslo. Sequence read demultiplexing was carried out using a custom routine developed at the Norwegian Sequencing Centre (https://github.com/nsc‐norway/triple_index‐demultiplexing). Further sequence data processing was performed using the Divisive Amplicon Denoising Algorithm as implemented in the *dada2* v1.16 R package (Callahan et al. 2016). Taxonomic classification of amplicon sequence variants (ASVs) was done using the SILVA v138.1 16S rRNA gene reference database (Quast et al. 2013). The read depth was adjusted through common scaling to the smallest read depth (28,430 reads) (McMurdie and Holmes 2014). Singletons as well as reads identified as either mitochondria or chloroplasts were removed from the data.

### 2.7 Statistical Analyses

All analyses were run with R (version 4.4.1). Diet diversity for each individual was calculated using sequence reads across MOTU's grouped into 14 families using Hill's numbers (Hill 1973; Keylock 2005) for *q* = 1 corresponding to the exponential of Shannon's entropy index (Spellerberg and Fedor 2003). Microbiome richness and diversity were estimated using Chao1 and Shannon's entropy indices. The inter‐annual variation in major components of both the diet (proportion of grasses and proportion of *Salix*) and the gut microbiome (Bacteroidota and Firmicutes phyla) was arcsine transformed and analysed using linear models. Year and valley were first fitted as categorical variables (degrees of freedom = 6 and 3, respectively). Linear trends over time (year as a linear variable instead of factor variable) or in one of the environmental variables reflecting plant productivity (maximum NDVI or July temperature) were tested in competing models, and the most parsimonious models were selected based on AIC value. Results from AIC model comparisons are presented in Table S3. The analysis of body mass in relation to the proportion of *Salix* in the diet was analysed using a generalised additive mixed model (function ‘gamm’ in the gamm4 package) with year as a random intercept. The fixed linear effects included the proportion of *Salix* in the diet, reproductive status (lactating, non‐lactating), valley, and two‐way interactions between *Salix* and lactating/not lactating, and also *Salix* and valley. Age was fitted with a spline function because of the curvilinear relationship with body mass.

Assessing the effects of valley, year, and the amount of *Salix* in the diet on the gut microbiome, PERMANOVA was carried out using the ‘*adonis2*’ functions with 10,000 permutations and Bray–Curtis distances in the *vegan* version 2.5.6 package. We set up the ‘by = terms’ setting, considering ‘valley’ as the first term, ‘year’ as the second term, and the amount of *Salix* in the diet as the third and last term, in order to account for the confounding effects of the year and valley. In order to further look for associations between diet and microbiome compositions, we used the ‘*mantel*’ function from the same R package. Partial redundancy analysis (RDA) was carried out using the *rda* function in the *vegan* package. The data matrix was transformed using the ‘hellinger’ option in the *decostand* function in *vegan*. In the model formulation, ‘valley’ and ‘year’ were included as conditioning variables in order to focus only on the effects of the proportion of *Salix* in the reindeer diet. Computation of *p*‐value for the *Salix* term in the RDA model was done using the *anova.cca* in *vegan* function and with 10,000 permutations. We carried out a non‐metric multidimensional scaling (NMDS) analysis using the *metaMDS* function and Bray‐Curtis distances in the *vegan* package using the default settings, and contour lines were added with the *ordisurf* function from the same package. Generalised additive models (GAMs) were computed with the *gam* function in the *mgcv* package (Wood 2011), using 5 degrees of freedom for the smooth terms. To separate any direct effect of *Salix* on body mass from a possible indirect effect via differences in the gut microbiome, a piecewise structural equation model was fitted with function ‘psem’ from the package *piecewiseSEM* (Lefcheck 2016). ANCOM‐BC analysis was carried out using the ‘ancombc2’ function from the ANCOM‐BC R package (version 2.8.1) using standard parameters except that the Benjamini‐Hochberg method was used for *p*‐value adjustment.

## 3 Results

### 3.1 October Diet

**FIGURE 1:**
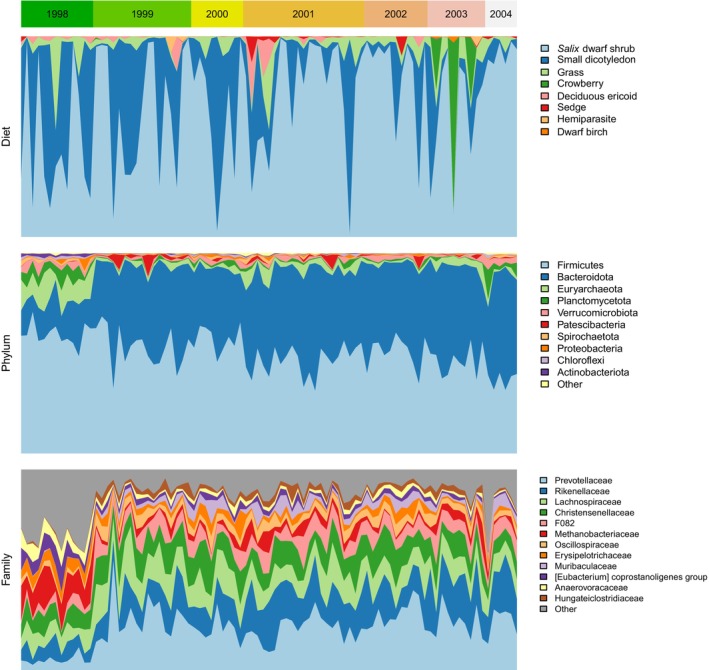
Temporal variation in the composition of the Svalbard reindeer diet (plant functional groups, upper panel) and the rumen microbiome (phylum and family level, lower two panels) over time. The width of the year bars is proportional to the sample size.

After bioinformatic analysis and data filtering, we retrieved 20,431,428 sequence reads from the samples passing all quality criteria. After removing data for calves and yearlings as well as failed samples, the final dataset comprised 97 individuals (Table S4). The Svalbard reindeer diet in October comprised 39 MOTUs from 14 different plant families. All plant MOTUs were identified at the family level, 33 (85%) at the genus level, and 13 MOTUs (33%) were identified at the species level (Table S5). The variation in RRA across the eight functional groups is illustrated in Figure 1a.

**FIGURE 2:**
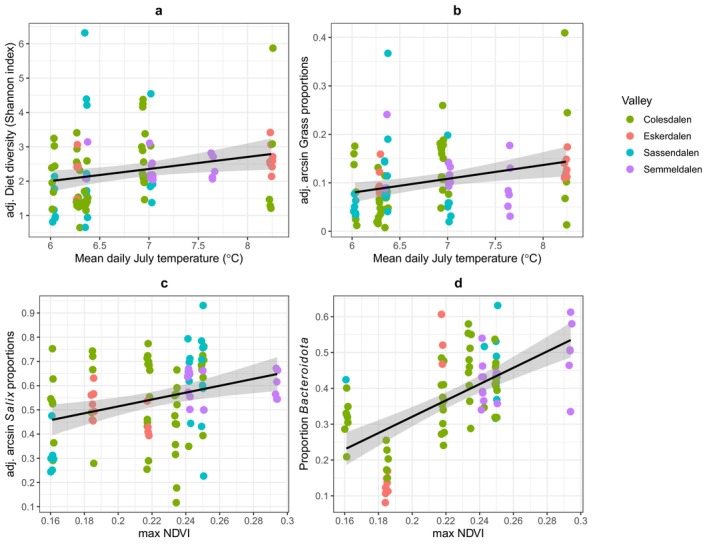
Diet diversity (Shannon index) of individual diets (a) and the adjusted arcsine RRA of grass in the reindeer diet (b) plotted against mean July temperature. The arcsine RRA of *Salix* in the diet (c) and of Bacteroidota, the main bacterial phylum within the Svalbard reindeer rumen microbiome, plotted against maximum NDVI (d). The *y*‐axis is adjusted for differences between valleys (coloured dots) and the points are the partial residuals.

Diet diversity at the family level differed significantly between valleys (*F*_3,92_ = 6.59, *p* < 0.001) and tended to increase over time (*F*_1,92_ = 3.03, *p* = 0.085). Diet diversity increased significantly following warmer summers (July temperature, *F*_1,92_ = 4.02, *p* = 0.048; Figure 2a). The RRA attributed to grasses was generally low (mean = 0.045 ± 0.068SD), and negatively correlated (*p* = 0.007) with the most common dietary item, the dwarf shrub *S. polaris* (mean = 0.639 ± 0.315SD), the only willow species on Svalbard. Differences between valleys in the grass RRA in the diet only tended towards significance (*p* = 0.068), but varied significantly between years (*F*_6,87_ = 3.77, *p* < 0.002). Grass RRA increased with mean July temperature (*p* = 0.015, Figure 2b) but not with maximum NDVI (*p* = 0.71). There were highly significant differences between valleys in the RRA of *Salix in the diet* (*F*_3,87_ = 30.8, *p* < 0.001), from least in Eskerdalen (0.245), Sassendalen (0.468), Colesdalen (0.745), to most in Semmeldalen (0.915) (Figure S3). After accounting for these valley differences, there was significant variation in the RRA of *Salix* in diet between years (*F*_6,87_ = 4.42, *p* < 0.001) but these showed no significant simple trend over time (*p* = 0.53). In contrast to grass, between‐year differences in *Salix* relative abundance were associated positively with maximum NDVI (*p* = 0.009, Figure 2c), but not with July temperature (*p* = 0.4).

### 3.2 Body Mass and Diet

**FIGURE 3:**
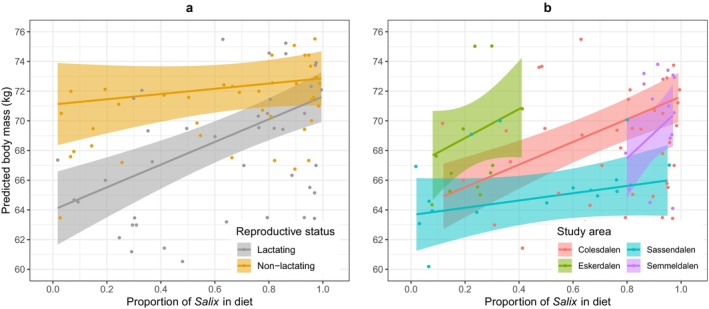
(a) The relationship between the adjusted October body mass of individuals (after accounting for Age and Valley: See Table S6), and the RRA of *Salix* dwarf shrub in their diet for lactating and non‐lactating females, and (b) for lactating females from each of the four valleys. The points are the partial residuals, adjusting for covariate effects. Age was fixed at 7 years; year was fixed at 2001, and location either was set to ‘Colesdalen’ (a) or varied across all study locations (b). This approach allows visualisation of individual variation in body mass while controlling for systematic differences associated with these covariates.

After accounting for effects of valley and age, October body mass was positively related to the *Salix* RRA in diet, but only for lactating females (Figure 3a, Table S6). While the predicted increase in mass was as high as 7.6 kg from a *Salix*‐free to an all‐*Salix* diet among lactating females, the equivalent estimate was 1.8 kg for non‐lactating females, with the interaction between reproductive status and *Salix* RRA close to significance (*p* = 0.055; Table S6). Despite differences in the average reindeer body mass between the valleys, among lactating females there was no significant interaction between *Salix* RRA in diet and valleys (Figure 3b, *p* = 0.75, Table S6).

### 3.3 The Gut Microbiome

For 87 of the 97 individuals used in the diet analysis, we also retrieved matching gut microbiome data. After quality filtering, we retained a total read number of 5,434,536 (mean: 62466; SD: 13831) matching 5040 ASVs. We identified 24 unique bacterial phyla within the reindeer rumen microbiome, with Bacteroidota (0.385 ± 0.13SD) and Firmicutes (0.428 ± 0.084SD) being the most abundant in terms of sequence reads (Figure 1b). More than 60% of bacterial ASVs could be assigned to the family level, with a total of 138 families identified (Figure 1c). Taxonomic classification accuracy of microbiome data is presented in Figure S4. The proportion of sequence reads assigned as Bacteroidota did not differ significantly between valleys (Wald statistic = 2.21, d.f. = 3, *p* = 0.41, Figure S6) but increased over the course of the study (Wald statistic = 12.21, d.f. = 1, *p* = 0.007, Figure 1). An equally plausible model is that the temporal trend reflects annual variation in max NDVI (Figure 2d, Table S3: delta AIC = 1.1). In contrast to Bacteroidota, Firmicutes differed between valleys (Eskerdalen = 0.379, Sassendalen = 0.382, Semmeldalen 0.445, Colesdalen = 0.457: Wald's statistic = 19.96, d.f. = 3, *p* < 0.001) and decreased significantly over the course of the study (Wald statistic = 21.97, d.f. = 1, *p* = 0.001). Despite the changes in Bacteroidota and Firmicutes, the overall diversity of the microbiome did not change over the course of the study.

### 3.4 Diet‐Microbiome Interactions

**FIGURE 4:**
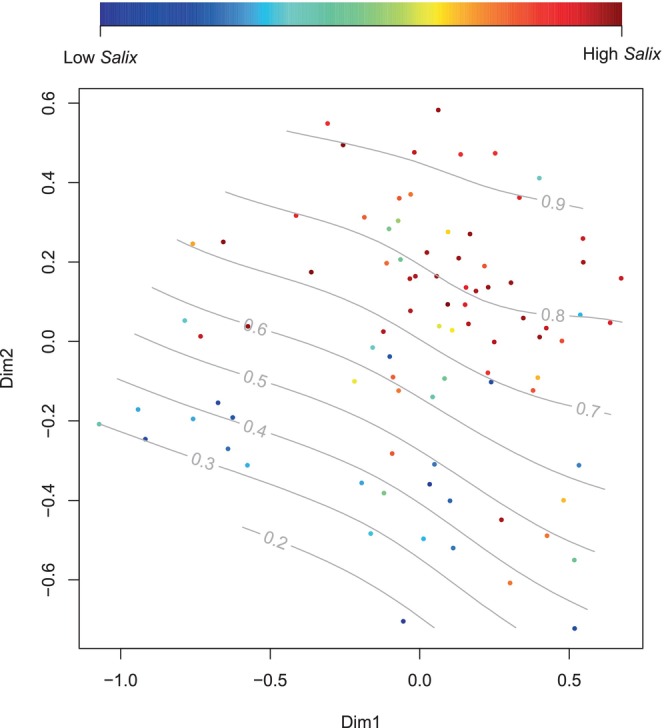
Non‐metric multidimensional scaling of the Bray–Curtis distance matrix of the Svalbard reindeer rumen microbiome composition according to the RRA of *Salix* dwarf shrub ( *Salix polaris* ) in the reindeer diet. The effect is significant (*p* = 0.012, PERMANOVA).

**FIGURE 5:**
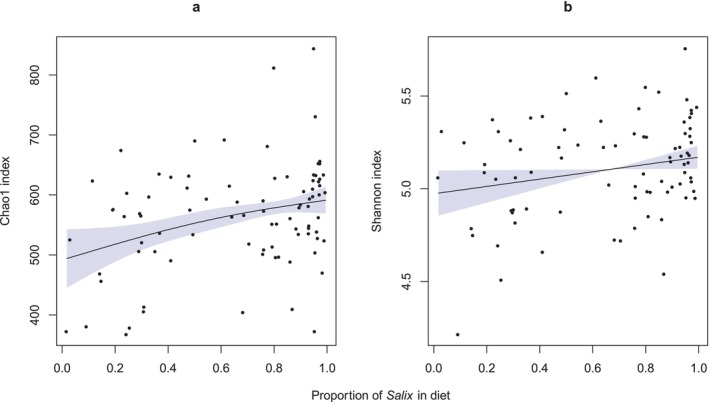
Generalised additive models showing the relationship between measures of microbiome diversity and the RRA of *Salix* in the diet of individual reindeer, with the shaded bands indicating 95% confidence limits of the models. Microbiome Chao1 index (*p* = 0.007) (a), and microbiome Shannon index (*p* = 0.039) (b).

Mantel testing demonstrated a highly significant relationship between diet and microbiome composition (Mantel *r* statistic: 0.27 using Pearson's correlation and 0.28 using Spearman's correlation, *p* < 0.001 for both tests, number of permutations 10,000). At the individual level, the RRA of *Salix* in the diet explained a significant amount of the variation in the gut microbiome composition (Figure 4, *p* = 0.012, *F*‐statistic: 1.62, *R* ^2^: 0.01, PERMANOVA), after accounting for the potential confounding effects of valley and year. The effect of dietary *Salix* on the rumen microbiome was also confirmed with a separate modelling approach using partial RDA (*p* = 0.0015). Moreover, both Shannon entropy and Chao1 microbiome diversity measures were significantly positively correlated with the proportion of sequence reads assigned to *Salix* in the diet (Figure 5).

The piecewise structural equation model indicated that the contribution of the direct path effect of *Salix* on body mass (partial correlation = 0.36 for Shannon; 0.41 for Chao1, after removing the effect of the significant correlation with the microbiome diversity) was greater than the indirect path effect through the microbiome diversity (0.29 × −0.26 = −0.08 for Shannon; 0.39 × −0.31 = −0.12 for Chao1) (Figure S6). Finally, after accounting for valley differences in diversity measures, there was no simple relationship between diet diversity and microbiome diversity (Shannon's entropy index: *b* = 0.011 ± 0.027SE, Chao1 index: *b* = −0.04 ± 8.8SE). ANCOM‐BC analysis did not identify any bacterial family‐level taxa significantly related to body mass (*p* > 0.5 after *p*‐value correction for all taxa).

## 4 Discussion

### 4.1 Body Mass, Diet and Interactions With Arctic Warming

An increase in plant biomass at a circumpolar scale is one of the major manifestations of climate warming in the Arctic tundra biome (Berner et al. 2020), potentially increasing carrying capacity for herbivore species. However, studies focusing on the impact of Arctic greening on large herbivores' foraging, fitness‐related traits, and population consequences remain rare. The sparse literature suggests both positive (Gagnon et al. 2020) and negative impacts on *Rangifer* (Fauchald et al. 2017). By combining multi‐year diet and gut microbiome DNA metabarcoding datasets, we demonstrate a significant positive correlation between the RRA of *S. polaris* , the major dwarf shrub on Svalbard, and gut bacterial composition and diversity. Also, higher RRA of *S. polaris* in diet significantly increased autumn body mass, an important fitness‐related trait. Annual variation in *Salix* RRA was significantly, positively related to the maximum NDVI, which we know reflects variation in primary productivity on Svalbard (Karlsen et al. 2018). Thus, our study suggests that when forage is plentiful, it is possible for reindeer to select diets composed of relatively more *Salix*, revealing a potentially important mechanism mediating the ecological impact of climate warming on body mass. Since autumn body mass has a strong influence on ovulation rates in Svalbard reindeer (Albon et al. 2017), recent climate warming and the associated higher plant biomass offer a likely explanation for the growth of Svalbard reindeer populations (Le Moullec et al. 2019).

A major finding of our study is that in October the heaviest animals were those that had higher RRA of *Salix* detected in their rumen. This relationship was particularly pronounced for females bearing the cost of lactation over the summer, compared to non‐lactating females. This could be explained by the fact that the energy cost of lactation in females rearing a calf leaves less scope for partitioning resources into their somatic recovery and effectively has less time to lay down fat (see also Chan‐McLeod et al. 1999). Access to *Salix* in the post‐weaning period in late summer and autumn seems to be important for body mass recovery, reducing the cost of reproduction. In comparison, non‐lactating females tend to be in good condition irrespective of *Salix* content in their October diet, suggesting that they replenished their fat stores earlier in the season.

Between‐year variation in the RRA of grass in the diet, but not *Salix*, was significantly related to July temperature as expected based on the temperature‐driven productivity measurements across plant functional types, recorded in the study area since 1998 (Van der Wal and Stien 2014). However, *Salix* RRA was significantly related to NDVI. Since we now know that annual variation in NDVI over a decadal time scale in our study area is strongly influenced by summer temperature (Karlsen et al. 2018, 2020), the lack of a direct correlation with July temperature may reflect that our 7‐year study is a relatively short time series for consistently detecting trends. For example, 1998, the first year in our study, was the warmest of the seven we had dietary measures from but had the second lowest maximum NDVI. Furthermore, independent studies on the annual growth of *Salix* found that productivity is not only dependent on July temperature but also negatively impacted by rain‐on‐snow events in the previous winter (Le Moullec et al. 2020). These rain‐on‐snow events are stochastic, vary in intensity, and also tend to be localised, which could well contribute to differences in annual *Salix* growth and hence availability between valleys. Finally, while above‐ground grass shoots are produced annually, shrub biomass accumulates over several years with a potential lagged positive effect of previous warm summers. This may contribute to explain why the dietary proportion of *Salix* was best explained by NDVI (capturing shrubification over time), while the proportion of grass was better explained by the current year's July temperature.

*Salix polaris* is a high‐quality resource for the Svalbard reindeer (Bjørkvoll et al. 2009; Le Moullec et al. 2020) due to its high abundance and relatively high nutritional quality (Van der Wal et al. 2000; Eikeland 2023). A significant proportion of the biomass of dwarf shrubs such as *Salix* is buried below‐ground but shallow enough in the soil to be accessible to herbivores after the above‐ground biomass has senesced (Iversen et al. 2015; Le Moullec et al. 2018). While there was a positive effect of *Salix* RRA in October on body mass, variation in *Salix* between individuals within years and valleys was large, suggesting this forage might not be equally available to reindeer. Svalbard reindeer have very small home ranges, within valleys, and the proportion of *Salix*‐rich habitat will vary at local scales, where topography and drainage will influence plant productivity during summer (Van der Wal and Stien 2014). Spatio‐temporal variation in plant senescence and impact of early snow in autumn can also potentially alter individuals' access to forage (Loe et al. 2020). Furthermore, since *Salix* RRA in the rumen likely corresponds to recent foraging (Barboza et al. 2006; Steuer et al. 2011; Picard et al. 2015), such diet ‘snapshot’ might not reflect the overall foraging strategy or preferences of individuals. This raises the question of the extent to which *Salix* is the ultimate driver of higher body mass or is representative of a more proximate overall foraging strategy depending on local conditions and individual energy requirements. Since autumn body mass in other *Rangifer* populations is also driven by summer foraging and weather conditions (e.g., heat stress that could reduce foraging time, Trondrud et al. 2023), we recognise the importance of longitudinal surveys to fully understand individual foraging strategies.

### 4.2 Gut Microbiome Variation and Interactions With Diet and Body Mass

Our results confirm the general understanding that dietary choice and gut microbiome composition are tightly linked (Muegge et al. 2011; Kartzinel et al. 2019; Baniel et al. 2021) as the characteristics of the dietary substrate determine which bacterial metabolic profiles will dominate the microbiome community. In our case, reindeer with high *Salix* RRA in the rumen displayed more similar gut microbiomes and higher microbiome diversity, suggesting a direct link with the foraging of this plant species by the reindeer. The higher microbiome diversity could potentially be explained by the fact that the less digestible autumn diet, especially the woody parts of *Salix*, available in October, can be retained in the gut for longer to maximise the extraction of nutrients, which in turn can increase gut microbiome diversity by favouring a variety of species specifically adapted to a high‐fibre diet, or enabling slow‐growing species to increase in numbers (McKenney et al. 2018; Reese and Dunn 2018).

Interestingly, we also observe a temporal trend in increasing Bacteroidota proportions in the reindeer rumen over time, correlating positively with NDVI. A similar increase in Bacteroidota proportions has been observed in Kalahari wild meerkats, where it correlated with extreme temperatures, disease, and poor body condition (Risely et al. 2023). Although our results do not allow us to pinpoint a direct link between the increase in Bacteroidota proportions and body mass, Svalbard reindeer numbers have been increasing over the course of the last 30 years, with the Nordenskiöld Land population more than doubling in abundance (Dwinnell et al. 2025). This suggests that within the high‐Arctic tundra ecosystem other mechanisms might be in play. Considering the strong relationship between diet and gut bacteria composition here as well as the relationship with NDVI, our findings open the question of whether the gradual change in microbiome composition over time could be considered indicative of a more general shift towards improved diet quality in the Svalbard reindeer over the last decade. Bacteroidota make important contributions to digestion, host metabolism, and overall health (Ley et al. 2008; Cholewińska et al. 2020), and their absolute abundances can increase with the increase in the supply of dietary nitrogen in the mammalian gut—an important limiting factor for both the host and the bacterial symbionts within the gut (Reese et al. 2018). During autumn in our study area, *S. polaris* is the plant with the highest nitrogen content (Eikeland 2023). Thus, if *S. polaris* has been increasingly accessible to reindeer in October (e.g., due to the warmer autumns and delay in onset of winter in Svalbard, Loe et al. 2020), this might have enabled upregulation by reindeer of preferred Bacteroidota via increased nitrogen supply to reinforce their positive effect on digestion and body mass (Foster et al. 2017).

Contrary to our expectations, and despite a significant relationship between *Salix* RRA and gut microbiome diversity, we did not detect a positive effect of gut microbiome diversity on reindeer body mass. Previous studies in wild populations such as the Seychelles warblers (Worsley et al. 2021) and the Sable Island feral horses (Stothart et al. 2024) show no relationship between microbial richness and diversity and fitness proxies, and suggest a leading role for bacterial community composition (and the associated gene families and metabolic functions) rather than bacterial diversity per se. The negative association between bacterial richness and reindeer body mass in our study is surprising and not necessarily easy to explain. It might reflect a balance between gaining nutritional benefits from *Salix* ingestion and the capacity of the gut microbiome to detoxify deterrent secondary compounds. Recent, independent measurements from the same study area carried out in 2022 do show that shrubs such as *Salix* and *Saxifraga* exhibit higher concentrations of phenolics (plant toxins typically acting as deterrents to herbivores) compared to forbs and grasses, and that these concentrations remain high even during the autumn (Eikeland 2023).

## 5 Conclusion

Our findings suggest a mechanistic understanding of how a keystone herbivore species has benefited from climate warming. There was a higher ingestion of *S. polaris* following summers of higher plant productivity (as indexed by the maximum NDVI). For reproductive females, a higher RRA of *Salix* in the autumn diet was associated with higher body mass, which in turn is known to improve future reproduction and overwinter survival. While we found a strong link between *Salix* intake and rumen bacterial composition, there was only a weak direct relationship between the gut microbiome and body mass. Our study highlights the value of combining fine‐grained DNA metabarcoding data to reveal the effect of diet–microbiome linkages on a fitness‐related trait and population growth of wild‐ranging ungulates. This approach is also highly relevant for addressing the importance of diet and gut microbiome adaptability in other herbivore populations challenged by climate warming.

## Author Contributions

**Stefaniya Kamenova:** conceptualization (equal), data curation (supporting), formal analysis (supporting), investigation (equal), methodology (equal), writing – original draft (lead). **Steve D. Albon:** conceptualization (equal), data curation (equal), formal analysis (equal), funding acquisition (supporting), investigation (equal), writing – original draft (supporting). **Leif Egil Loe:** conceptualization (equal), data curation (equal), formal analysis (equal), funding acquisition (lead), investigation (equal), project administration (lead), writing – original draft (supporting), writing – review and editing (supporting). **R. Justin Irvine:** conceptualization (equal), investigation (equal), methodology (equal), resources (equal), writing – review and editing (supporting). **Rolf Langvatn:** investigation (supporting), methodology (supporting), resources (supporting). **Galina Gusarova:** conceptualization (supporting), funding acquisition (equal), methodology (supporting), project administration (supporting), resources (equal). **Eric Jacques de Muinck:** data curation (supporting), funding acquisition (equal), investigation (supporting), methodology (equal), resources (equal). **Pål Trosvik:** conceptualization (equal), data curation (equal), formal analysis (lead), funding acquisition (equal), investigation (equal), methodology (equal), writing – review and editing (supporting).

## Disclosure

Permits: Reindeer sample collection met all guidelines of the Norwegian Food Safety Authority (current permit number 24/4546) and samples were collected with the permission of the Governor of Svalbard (current permit number 21/03815‐23).

## Conflicts of Interest

The authors declare no conflicts of interest.

## Supporting information


Appendix S1.

## Data Availability

Raw sequencing datasets are available at the Sequence Read Archive database under accession numbers PRJNA1052483 (DNA metabarcoding diet data) and PRJNA1044740 (16S amplicon microbiome data). All the relevant data files, metadata and scripts are publicly available as a Figshare project “Climate‐diet interactions impact reindeer body mass” and can be accessed via the link: https://figshare.com/projects/Climate‐diet_interactions_impact_reindeer_body_mass/223470.
