# Supplementary material for: Arctic Greening Drives Changes in the Diet and Gut Microbiome of a Large Herbivore With Consequences for Body Mass
Source: Ecol Evol. 2025 Jul 22;15(7):e71731. doi: 10.1002/ece3.71731 (PMC12281024; doi:10.1002/ece3.71731)
Supplement: Supplementary file 1 — Appendix S1. [file ECE3-15-e71731-s001.docx]

**Supplemental information**

**Title:** Arctic greening drives changes in the diet and gut microbiome of a large herbivore with consequences for body mass

**Table S1.** Habitat classification and percentage of vegetation cover for each of the four valleys. Estimates are based on data from Johansen BE, Karlsen SR, Tømmervik H (2012) Vegetation mapping of Svalbard utilising Landsat TM/ETM+ data. Polar Record 48, 47–63. DOI:10.1017/S0032247411000647

| Valley | % Grass sward | % *Luzula* heath | % Marsh | % Ridge | % Other | % Not classified |
| --- | --- | --- | --- | --- | --- | --- |
| Colesdalen | 9,6 | 17,7 | 3,5 | 4,4 | 57,0 | 7,8 |
| Eskerdalen | 14,3 | 22,7 | 0,4 | 4,1 | 56,0 | 2,5 |
| Sassendalen | 6,6 | 13,1 | 7,1 | 7,7 | 58,3 | 7,2 |
| Semmeldalen | 19,4 | 36,5 | 0,3 | 2,8 | 39,1 | 1,8 |

**Table S2.** Details about the six synthetic DNA standards used as a positive control mock community, amplified with the *Sper01* seed plant primers as part of the DNA metabarcoding diet analysis of the Svalbard reindeer.

| Synthetic standard | Dilution | Reference sequence | Standard length (bp) | Standard GC content (%) |
| --- | --- | --- | --- | --- |
|  |  |  |  |  |
| SS_01_GH | 1 | taagtctcgcactagttgtgacctaacgaatagagaattctataagacgtgttgtcccat | 60 | 40 |
| SS_02_GH | 1/2 | gtgtatggtatatttgaataatattaaatagaatttaatcaatctttacatcgcttaata | 60 | 20 |
| SS_03_GH | 1/4 | cacaatgctcggtaactagaagcatttgta | 30 | 40 |
| SS_04_GH | 1/8 | attgaatgaaaagattattcgatatagaat | 30 | 20 |
| SS_05_GH | 1/16 | agaacgctagaatctaagatggggggggggatgagtaagatatttatcagtaacatatga | 60 | 40 |
| SS_06_GH | 1/32 | atttttgtaactcattaacaattttttttttgatgtatcataagtactaaactagttact | 60 | 20 |

**Table S3.** Model selection based on AIC of linear models (LM) assessing inter-annual variation in major components of both the Svalbard reindeer gut microbiome (Bacteroidota or Firmicutes phyla) and diet (proportion of grasses and proportion of *Salix*). NDVI corresponds to the maximum normalised difference vegetation index, while JJT corresponds to July temperature.

| **Response variable** | **Model** | **Predictors** | **df** | **AIC** | **ΔAIC** |
| --- | --- | --- | --- | --- | --- |
| Firmicutes | f2 | Location + Year (linear) | 6 | **–225.1** | 0.0 |
|  | f1 | Location + Year (factor) | 11 | –223.0 | 2.1 |
|  | f3 | Location + NDVI | 6 | –215.9 | 9.2 |
|  | f4 | Location + JJT | 6 | –197.3 | 27.8 |
|  | f0 | Intercept only | 2 | –182.9 | 42.2 |
| Bacteroidetes | b1 | Location + Year (factor) | 11 | **–183.8** | 0.0 |
|  | b2 | Location + Year (linear) | 6 | –141.4 | 42.4 |
|  | b3 | Location + NDVI | 6 | –140.3 | 43.5 |
|  | b4 | Location + JJT | 6 | –126.8 | 57.0 |
|  | b0 | Intercept only | 2 | –92.2 | 91.6 |
| Grass | g1 | Location + Year (factor) | 12 | **–135.6** | 0.0 |
|  | g4 | Location + JJT | 7 | –130.1 | 5.5 |
|  | g0 | Intercept only | 2 | –125.9 | 9.7 |
|  | g2 | Location + Year (linear) | 7 | –125.6 | 10.0 |
|  | g3 | Location + NDVI | 7 | –124.8 | 10.8 |
| *Salix* | s1 | Location + Year (factor) | 12 | **21.4** | 0.0 |
|  | s3 | Location + NDVI | 7 | 30.0 | 8.6 |
|  | s4 | Location + JJT | 7 | 34.1 | 12.6 |
|  | s2 | Location + Year (linear) | 7 | 36.6 | 15.2 |
|  | s0 | Intercept only | 2 | 92.1 | 70.7 |

**Table S4.** Number of individual Svalbard reindeer rumen samples per year and locality retained in the final diet-gut microbiome matched dataset.

|  |  |  |  |  |
| --- | --- | --- | --- | --- |
|  | Colesdalen | Eskerdalen | Sassendalen | Semmeldalen |
|  |  |  |  |  |
| 1998 | 7 | 8 | 0 | 0 |
| 1999 | 13 | 5 | 0 | 0 |
| 2000 | 7 | 0 | 2 | 0 |
| 2001 | 10 | 0 | 10 | 2 |
| 2002 | 1 | 0 | 1 | 9 |
| 2003 | 10 | 0 | 0 | 0 |
| 2004 | 0 | 0 | 0 | 8 |

**Table S5.** Overall plant diet composition of the Svalbard reindeer assessed with DNA metabarcoding from 97 rumen content samples, collected in October between 1998 and 2004.

|  |  |  |  |  |  |  |
| --- | --- | --- | --- | --- | --- | --- |
| **Best identity match** | **Family** | **Genus** | **Species** | **Scientific name** | **Assumed taxon** | **Functional category** |
|  |  |  |  |  |  |  |
| 1 | Salicaceae | NA | NA | Saliceae | Salix sp. | Salix dwarf shrub |
| 1 | Saxifragaceae | *Saxifraga* | *Saxifraga oppositifolia* | *Saxifraga oppositifolia* | *Saxifraga oppositifolia* | Small dicotyledon |
| 1 | Poaceae | NA | NA | Pooideae | Poaceae | Grass |
| 1 | Poaceae | *Festuca* | NA | *Festuca* | *Festuca* sp. | Grass |
| 1 | Polygonaceae | *Bistorta* | *Bistorta vivipara* | *Bistorta vivipara* | *Bistorta vivipara* | Small dicotyledon |
| 0.98 | Saxifragaceae | *Saxifraga* | *Saxifraga oppositifolia* | *Saxifraga oppositifolia* | *Saxifraga oppositifolia* | Small dicotyledon |
| 1 | Rosaceae | *Dryas* | NA | *Dryas* | *Dryas octopetala* | Deciduous ericoid |
| 1 | Ericaceae | *Empetrum* | NA | *Empetrum* | *Empetrum nigrum* | Crowberry |
| 1 | Brassicaceae | *Draba* | NA | *Draba* | *Draba* sp. | Small dicotyledon |
| 1 | Saxifragaceae | *Saxifraga* | NA | *Saxifraga* | *Saxifraga* sp. | Small dicotyledon |
| 1 | Saxifragaceae | *Saxifraga* | *Saxifraga cespitosa* | *Saxifraga cespitosa* | *Saxifraga cespitosa* | Small dicotyledon |
| 1 | Polygonaceae | *Oxyria* | *Oxyria digyna* | *Oxyria digyna* | *Oxyria digyna* | Small dicotyledon |
| 1 | Poaceae | NA | NA | Poinae | Poaceae | Grass |
| 1 | Betulaceae | *Betula* | NA | *Betula* | *Betula nana* | Dwarf birch |
| 0.96 | Saxifragaceae | *Saxifraga* | NA | *Saxifraga* | *Saxifraga* sp. | Small dicotyledon |
| 1 | Poaceae | NA | NA | Poeae | Poaceae | Grass |
| 1 | Caryophyllaceae | *Cerastium* | NA | *Cerastium* | *Cerastium* sp. | Small dicotyledon |
| 0.98 | Saxifragaceae | *Saxifraga* | NA | *Saxifraga* | *Saxifraga* sp. | Small dicotyledon |
| 1 | Juncaceae | *Luzula* | NA | *Luzula* | *Luzula* sp. | Sedge |
| 0.98 | Salicaceae | NA | NA | Saliceae | *Salix* sp. | Salix dwarf shrub |
| 1 | Ranunculaceae | *Ranunculus* | Ranunculus pygmaeus | *Ranunculus pygmaeus* | *Ranunculus pygmaeus* | Small dicotyledon |
| 1 | Orobanchaceae | *Pedicularis* | NA | *Pedicularis* | *Pedicularis* sp. | Hemiparasite |
| 1 | Poaceae | NA | NA | Poeae | Poaceae | Grass |
| 1 | Papaveraceae | *Papaver* | NA | *Papaver* | *Papaver* sp. | Small dicotyledon |
| 1 | Caryophyllaceae | *Minuartia* | *Minuartia biflora* | *Minuartia biflora* | *Minuartia biflora* | Small dicotyledon |
| 1 | Ericaceae | *Cassiope* | *Cassiope tetragona* | *Cassiope tetragona* | *Cassiope tetragona* | Deciduous ericoid |
| 0.98 | Ericaceae | *Cassiope* | *Cassiope tetragona* | *Cassiope tetragona* | *Cassiope tetragona* | Deciduous ericoid |
| 0.97 | Ranunculaceae | *Ranunculus* | *Ranunculus pygmaeus* | *Ranunculus pygmaeus* | *Ranunculus pygmaeus* | Small dicotyledon |
| 0.98 | Rosaceae | *Dryas* | NA | *Dryas* | *Dryas octopetala* | Deciduous ericoid |
| 1 | Orobanchaceae | *Pedicularis* | NA | *Pedicularis* | *Pedicularis* sp. | Hemiparasite |
| 1 | Poaceae | *Puccinellia* | NA | *Puccinellia* | *Puccinellia* sp. | Grass |
| 1 | Cyperaceae | *Carex* | NA | *Carex* | *Carex* sp. | Sedge |
| 0.96 | Ericaceae | *Cassiope* | *Cassiope tetragona* | *Cassiope tetragona* | *Cassiope tetragona* | Deciduous ericoid |
| 1 | Cyperaceae | *Eriophorum* | NA | *Eriophorum* | Eriophorum sp. | Sedge |
| 1 | Saxifragaceae | *Saxifraga* | *Saxifraga hirculus* | *Saxifraga hirculus* | *Saxifraga hirculus* | Small dicotyledon |
| 1 | Saxifragaceae | *Saxifraga* | NA | *Saxifraga* | *Saxifraga* sp. | Small dicotyledon |
| 1 | Orobanchaceae | *Pedicularis* | *Pedicularis lanata* | *Pedicularis lanata* | *Pedicularis lanata* | Hemiparasite |
| 1 | Rosaceae | *Potentilla* | NA | *Potentilla* | *Potentilla* sp. | Small dicotyledon |
| 0.98 | Saxifragaceae | *Saxifraga* | NA | *Saxifraga* | *Saxifraga* sp. | Small dicotyledon |

**Table S6.** A generalised additive mixed model explaining variation in body mass of female Svalbard reindeer as a function of study area, proportion of *Salix* in the diet and lactation status, including two-way interactions. Age is accounted for in a separate non-linear spline term (estimated degrees of freedom = 4.4; p < 0.001) while year is fitted as a random intercept (with SD = 1.96). Note that there is a significant positive effect of *Salix* proportion on body mass (Estimate = 7.6, SE = 3.1, p = 0.016), indicating that higher *Salix* consumption is associated with greater body mass in lactating females. There is a marginally non-significant interaction between *Salix* proportion and lactation status (Estimate = -5.9, SE = 3.0, p = 0.055). This suggests a potential moderating effect of lactation status on the relationship between *Salix* consumption and body mass, in the direction that Salix is less important for non-lactating females. Although the effect of *Salix* does not differ significantly between valleys, we retained Valley in the model to account for imbalanced sampling across years and valleys. In conclusion the model reveals that the proportion of *Salix* in the diet and lactation status are significant determinants of body mass in female Svalbard reindeer, while the study area and the interaction effects do not show significant influence. The significant non-linear effect of age and the random effect of year underscore the importance of accounting for these factors in the analysis.

|  | **Estimate** | **SE** | **t** | **P** |
| --- | --- | --- | --- | --- |
| Intercept | 62.7 | 1.8 | 35.4 | <0.001 |
| *Salix* | 5.7 | 2.3 | 2.5 | 0.013 |
| Lactating (no vs yes) | 7.2 | 2.1 | 3.4 | 0.0009 |
| *Salix* x Lactating | -4.5 | 2.9 | -1.5 | 0.127 |

*
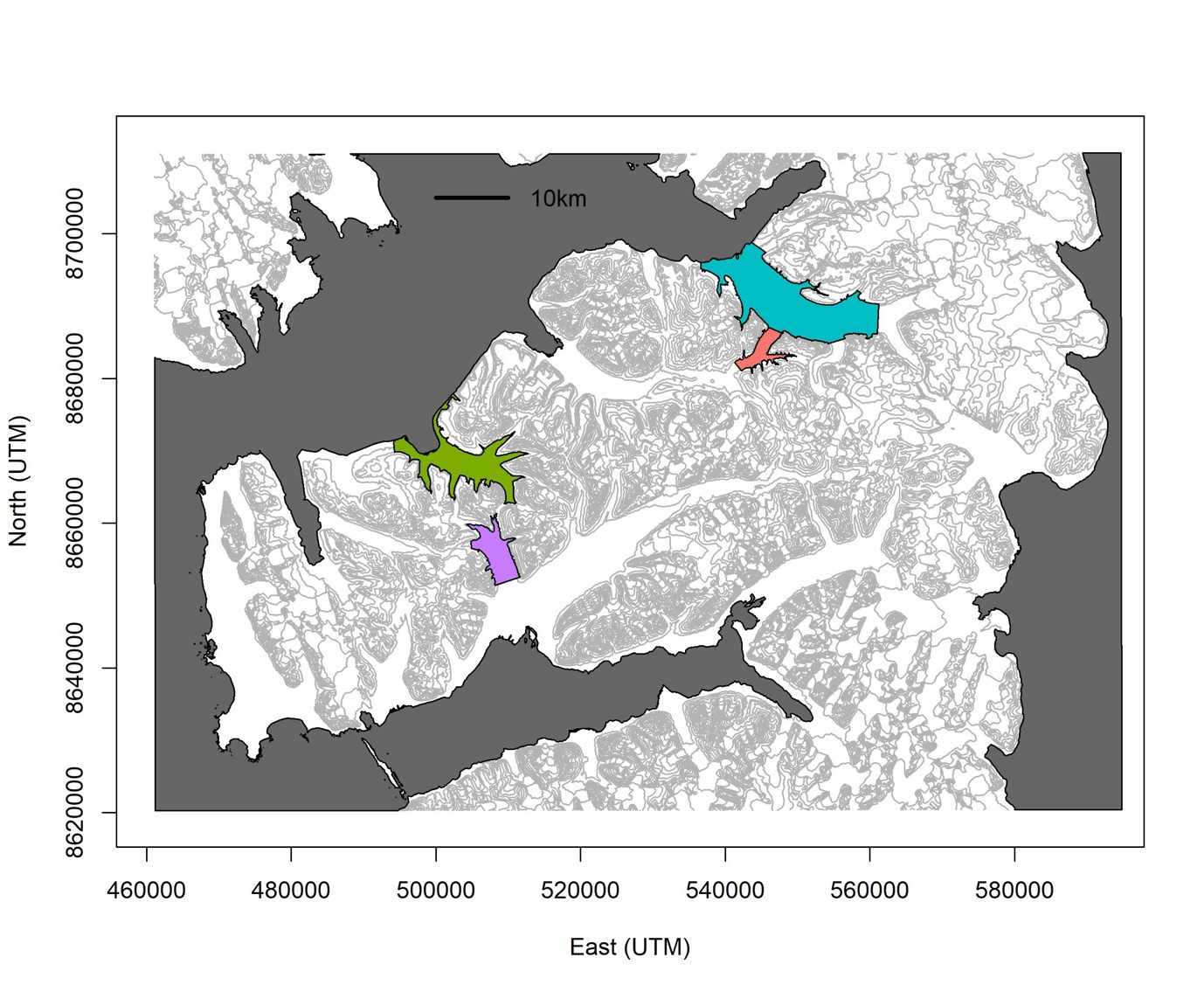
*

**Figure S1.** Map of the study area indicating the locations of the Colesdalen (green), Semmeldalen (purple), Eskerdalen (red) and Sassendalen (blue) valleys, and the main settlement Longyearbyen.

**Figure S2.** Relationships between dietary proportions for *Salix* and grasses estimated with DNA metabarcoding (relative sequence reads abundance, protocols detailed in this study) and micro-histological examination of digested plant fragments in rumen (protocols detailed in Bjørkvoll et al. 2009, DOI: 10.1657/1523-0430-41.1.88). Dietary estimates are correlated in each valley for both *Salix* (left-hand panel: Colesdalen (red triangles), r = 0.582, p < 0.001; Semmeldalen (blue diamonds), r = 0.479, p = 0.061) and Grass (right-hand panel: Colesdalen (red triangles), r = 0.581, p < 0.001; Semmeldalen (blue diamonds), r = 711, p = 0.002). For both *Salix* and Grass there is no difference in the slopes between the valleys (*Salix*.valley interaction, P = 0.596; Grass.valley interaction, p = 0.345).

**Figure S3.** Variation over time of the relative proportion of the different plant functional groups, detected in the October diet of the Svalbard reindeer for each year and sampling location.

**Figure S4.** Percentage of Amplicon Sequence Variants (ASVs) classified to the indicated taxonomic level with >50% probability for the Svalbard reindeer rumen microbiome dataset.

**Figure S5.** Rumen microbiome composition of the Svalbard reindeer, sampled in October between 1998 and 2004 across the four different valleys in Svalbard, Colesdalen, Eskerdalen, Sassendalen and Semmeldalen.

**Figure S6.** A piecewise structural equation model showing the direct and indirect (through microbiome) effect of *Salix* proportion in diet on age-specific body mass for lactating females. The values on the arrows are the standardized path coefficient and are effectively partial correlation coefficients, accounting for the correlation with the third variable. Arrow width is proportional to the strength of the coefficient**.**
